# Statistical Mechanics at Strong Coupling: A Bridge between Landsberg’s Energy Levels and Hill’s Nanothermodynamics

**DOI:** 10.3390/nano10122471

**Published:** 2020-12-10

**Authors:** Rodrigo de Miguel, J. Miguel Rubí

**Affiliations:** 1 Department of Teacher Education, Norwegian University of Science and Technology, 7491 Trondheim, Norway; 2 Department of Condensed Matter Physics, University of Barcelona, 08007 Barcelona, Spain; mrubi@ub.edu

**Keywords:** thermodynamics at strong coupling, *temperature-dependent* energy levels, thermodynamics of small systems, nanothermodynamics

## Abstract

We review and show the connection between three different theories proposed for the thermodynamic treatment of systems not obeying the additivity ansatz of classical thermodynamics. In the 1950s, Landsberg proposed that when a system comes into contact with a heat bath, its energy levels are redistributed. Based on this idea, he produced an extended thermostatistical framework that accounts for unknown interactions with the environment. A decade later, Hill devised his celebrated *nanothermodynamics*, where he introduced the concept of *subdivision potential*, a new thermodynamic variable that accounts for the vanishing additivity of increasingly smaller systems. More recently, a thermostatistical framework *at strong coupling* has been formulated to account for the presence of the environment through a Hamiltonian of mean force. We show that this modified Hamiltonian yields a temperature-dependent energy landscape as earlier suggested by Landsberg, and it provides a thermostatistical foundation for the subdivision potential, which is the cornerstone of Hill’s nanothermodynamics.

## 1. Introduction

Systems are always in contact with an environment that influences their energy, volume, and mass. In certain cases, the presence of the environment is of little significance and, for simplicity, the system may be described as though it were isolated. In other cases, surroundings significantly affect the properties of systems, and external interactions need to be taken into account. Systems subject to the latter scenario may be referred to as small, where *small* is not an attribute determined by the system’s sheer size, but rather by how the size compares to the range of the interactions affecting the system [1,2,3].

In contrast to their macroscopic analogues, small systems are *non-extensive*, and consequently, also *non-additive*, i.e., the total system is not just a sum of its parts. Clearly, this lack of additivity escapes the paradigms of classical thermodynamics, and a modified thermophysical theory is needed to describe small systems.

In 1940, Rushbrooke proposed a novel statistical mechanical method to account for the non-additivity of a system whose interactions with the environment are unknown [4]. This method, which consisted on allowing the energy levels of the system to depend on the temperature of the bath, was later refined by Landsberg in the 1950s [5,6]. In the decades that followed, Landsberg’s theory of *temperature-dependent* energy levels was directly invoked to study the temperature dependence of energy gaps in semiconductors [7], as well as bosonic systems where the standard thermodynamic description breaks down due to strong interactions [8,9]. Temperature-dependent energy levels have since become ubiquitous in the study of semiconductors (see e.g., [10,11] and references therein) and bosonic systems (see e.g., [12] and references therein). The theory has also been applied to superconductivity [13], optomechanical oscillators [14], irreversible effects in thermoelectric phenomena [15,16], small-system thermalization [17], and even information theory [18].

Despite the many applications of Landsberg’s framework, nobody used it to develop an alternative thermodynamic theory for nonadditive systems. In fact, the first thermodynamic theory valid for nonadditive systems did not appear until 1962, when Hill independently proposed his celebrated thermodynamics of small systems [19,20], later termed *nanothermodynamics* [21,22,23]. In contrast to Landsberg’s approach, Hill’s thermodynamic theory is not based on modified energy levels, but rather on the introduction of a *subdivision potential* to account for nonadditive effects. Hill’s theory has proven fruitful to describe nanoscale phenomena [24,25,26,27,28,29], and also the nonadditivity that occurs in macroscopic systems subject to long-range interactions [2].

In recent times, Landsberg’s idea of a statistical mechanics with temperature-dependent energy levels has covertly reappeared in a framework known as statistical mechanics *at strong coupling* [30]. This framework is based on the formulation of a *Hamiltonian of mean force*, which accounts for environmental perturbations on the small system of interest [30]. This thermostatistical approach has recently been applied to describe nanomechanical systems [31], nanoscale interfacial phenomena [3] and the advent of negative thermophoresis [32].

In this work, we show that Landsberg’s physical theory of temperature-dependent energy levels [6] is the foundation of the Hamiltonian of mean force upon which the recent statistical mechanics at strong coupling [30] is based. Furthermore, we show that statistical mechanics at strong coupling provides a thermostatistical foundation for the subdivision potential, which, as we shall see below, is the cornerstone of Hill’s nanothermodynamics [20]. Although the inception and evolution of these three powerful theories has been completely independent, and despite their apparent differences, they are indeed indeed deeply connected.

The remainder of this paper is structured as follows. In Section 2, we show that Landsberg’s theory of temperature-dependent energy levels lies at the heart of the Hamiltonian of mean force upon which statistical mechanics at strong coupling is based. In Section 3, we show that statistical mechanics at strong coupling provides a thermostatistical basis for the subdivision potential, the thermodynamic variable upon which Hill’s nanothermodynamics is based. Concluding remarks are given in Section 4.

## 2. ‘Temperature-Dependent’ Energy Levels and the Hamiltonian of Mean Force

In 1940, Rushbrooke proposed a generalized *statistical mechanics for assemblies whose energy-levels depend on the temperature*. His idea was that *the physical state of either part of the assembly will depend on the temperature, and thus so will its interaction with the other part of the assembly* [4]. Rushbrooke’s treatment was further generalized by Landsberg in the 1950s, when he applied the method to explore the equilibrium properties of systems subject to unknown interactions with some environment [5,6]. In the following, we show that Landsberg’s theory leads to the Hamiltonian of mean force used in statistical mechanics at strong coupling [30].

Landsberg’s theory may be summarized as follows. Considering an ensemble of systems at temperature *T*, one may abstractly decompose each system into two interacting parts: part 1 in state $i_{1}$ and with energy $E_{1}\left(i_{1}\right)$; and part 2 in state $i_{2}$ and with energy $E_{2}\left(i_{2}\right)$. It is then assumed that, while the effective energy of part 2 does not deviate from its isolated value, the interaction between both subsystems does affect the energy of part 1. The total energy of the interacting system may then be written as

(1) $$E\left(i_{1},i_{2}\right)=E_{1}\left(i_{1},i_{2}\right)+E_{2}\left(i_{2}\right),$$

and the effective energy levels $E_{1}\left(i_{1}\right)$ of part 1 become (see § 4 in [6])

(2) $$E_{1}\left(i_{1}\right)=-k_{B}T\ln\left[\frac{\sum_{i_{2}}e^{-E_{1}\left(i_{1},i_{2}\right)/k_{B}T}\;e^{-E_{2}\left(i_{2}\right)/k_{B}T}}{\sum_{i_{2}}e^{-E_{2}\left(i_{2}\right)/k_{B}T}}\right].$$

This method is useful when there is incomplete statistical mechanical information, and the interactions between the system (part 1) and the environment (part 2) are unknown. The modified energy levels $E_{1}\left(i_{1}\right)$ are anomalous in that they are effectively temperature-dependent. Inserting these effective energy levels into the partition function

(3) $$Z_{1}=\sum_{i_{1}}e^{-E_{1}\left(i_{1}\right)/k_{B}T}$$

results in effective expressions for the internal energy $U_{1}$ and the entropy $S_{1}$ of the system

(4) $$U_{1}=k_{B}T^{2}\frac{\partial }{\partial T}\log Z_{1}=\langle E_{1}\left(i_{1}\right)\rangle -T\langle \frac{dE_{1}\left(i_{1}\right)}{dT}\rangle ,$$

(5) $$S_{1}=\frac{U_{1}}{T}+k_{B}\ln Z_{1}=\frac{\langle E_{1}\left(i_{1}\right)\rangle }{T}+k_{B}\ln Z_{1}-\langle \frac{dE_{1}\left(i_{1}\right)}{dT}\rangle ,$$

which differ from the usual additive expressions by the incidence of the last terms (see § 2 in [6]).

It should be noted that, in the approximation in which interactions are neglected, then $E_{1}\left(i_{1},i_{2}\right)$ simply becomes $E_{1}\left(i_{1}\right)$, and the temperature-dependent energy levels $E_{1}\left(i_{1}\right)$ reduce to the temperature-independent, purely mechanical energies $E_{1}\left(i_{1}\right)$. In general, however, temperature-dependence does emerge into effective energy levels. Indeed, when examining energy levels in polarons, Whitfield and Engineer found two distinct types of temperature-dependence: while the temperature-dependent levels $E_{1}\left(i_{1}\right)$ in the partition function (15) determine the probability that state $i_{1}$ is occupied, the $E_{1}\left(i_{1}\right)-T\;d_{T}E_{1}\left(i_{1}\right)$ averaged in (4) determine the contribution actually made to the total energy by that state when it is occupied [33]. Incidentally, Landsberg’s method of temperature-dependent energy levels is briefly mentioned in Pathria and Beale’s landmark tektbook on statistical mechanics (see ch. 3, footnote 1 in [34]).

In recent years, and independently of Landsberg’s work, a *statistical mechanics and thermodynamics at strong coupling* has been developed on the basis of a *Hamiltonian of mean force* [30,35,36,37]. The Hamiltonian of mean force is indeed a special case of (1), where the Hamiltonian of $E_{1}\left(i_{1},i_{2}\right)$ may be decomposed as $H_{1}\left(i_{1},i_{2}\right)=H_{1}\left(i_{1}\right)+I\left(i_{1},i_{2}\right)$, such that the total Hamiltonian $H(i_{1},i_{2})$ is expressed as a sum of three terms:

(6) $$H\left(i_{1},i_{2}\right)=H_{1}\left(i_{1}\right)+I\left(i_{1},i_{2}\right)+H_{2}\left(i_{2}\right).$$

The terms $H_{1}$ and $H_{2}$ correspond, respectively, to the bare system and the bare environment, and I is the ineraction Hamiltonian between the system and the enviroment. Averaging $H_{1}$ and I over the bare environment $H_{2}$ results in a temperature-dependent Hamiltonian $H_{1}$ of mean force for the system given by [30]:

(7) $$H_{1}\left(i_{1}\right)=-k_{B}T\ln\left[\frac{\sum_{i_{2}}e^{-\left\{H_{1}\left(i_{1}\right)+I\left(i_{1},i_{2}\right)\right\}/k_{B}T}e^{-H_{2}\left(i_{2}\right)/k_{B}T}}{\sum_{i_{2}}e^{-H_{2}\left(i_{2}\right)/k_{B}T}}\right].$$

In a regime where the interactions I with the environment are negligible compared to the bare system’s energy $H_{1}$, the Hamiltonian of mean force $H_{1}$ reduces to the bare (and temperature-independent) $H_{1}$. In the following section, we show that the thermostatistical theory *at strong coupling* developed on the basis of this Hamiltonian of mean force [30] reproduces the subdivision potential proposed by Hill in his thermodynamics of small systems [20].

## 3. Strong Coupling and Hill’s Nanothermodynamics

In this section, we show that statistical mechanics at strong coupling provides a thermostatistical basis to Hill’s nanothermodynamics. We start by providing a modest introduction to Hill’s thermodynamic theory. We then use statistical mechanics at strong coupling to show that the subdivision potential in Hill’s theory is reproduced when one considers the temperature-dependence of the effective Hamiltonian which results from the interaction between the system and the environment.

### 3.1. Hill’s Subdivision Potential

In his nanothermodynamics [20], Hill starts by considering a traditional, homogeneous thermodynamic system with temperature *T*, pressure *p* and chemical potential μ. As this system is abstractly divided into smaller and smaller identical subsystems, the internal energy of each subsystem becomes eventually comparable to the energy of interaction amongst the subsystems, and, in contrast to traditional additive thermodynamics, the interaction can no longer be neglected. A new pair of conjugate variables emerges, namely the amount M of identical subsystems, and the *subdivision potential*E. This subdivision potential may be thought of as the difference between a subsystem’s true internal energy and the energy it would have if the rest of the subsystems were absent. For sufficiently large subsystems, the subdivision potential E is negligible.

After the subdivision process, each of the M identical subsystems has internal energy U, entropy S, volume V and N particles. Accounting for each subsystem’s subdivision potential E, the total energy of the system is given by a modified Euler equation:

(8) MU=TMS−pMV+μMN+ME.

And, for each individual subsystem, the energy becomes

(9) U=U+E,

with *U* given by the usual Euler expression

(10) U=TS−pV+μN.

Due to the incidence of the last term in (9), the internal energy U ceases to be a linear homogeneous function of S, V and N. This extra term, the subdivision potential E, is the cornerstone of Hill’s nonadditive nanothermodynamics. Paraphrasing Hill: *small-system thermodynamics departs from macroscopic thermodynamics in that [U]* *is not a linear homogeneous function of [S, V and N]*. *Hence and extra term occurs in (9)*. *This last two sentences epitomize the whole book* (see p. 24 in [20]).

When the subsystem of interest is an open system with a definite chemical potential μ and temperature *T* (imposed by the heat and particle reservoir made up of all other subsystems), then the additional energy term E in (9) must stem from an alteration in pressure. Interactions with the environment cause the system’s pressure to deviate from *p* and become instead an effective pressure $\hat{p}$. Then, the system’s internal energy is changed by an amount (see pp. 10, 24 in [20])

(11) $$E=-\left(\hat{p}-p\right)V.$$

If the subsystem of interest is, instead, a closed system with a definite pressure *p* and temperature *T*, then the additional energy must result from an alteration in chemical potential. Interactions with the environment cause the system’s chemical potential to deviate from μ and become instead an effective $\hat{\mu }$. Then, the system’s internal energy is altered by an amount (see pp. 16, 24 in [20])

(12) $$E=\left(\hat{\mu }-\mu \right)N.$$

If the subsystem’s only environmental constraint is the temperature *T*, then interactions with the environment will cause deviations in pressure (which becomes $\hat{p}$ instead of *p*) and chemical potential (which becomes $\hat{\mu }$ instead of μ). As a result, the additional subdivision energy E becomes

(13) $$E=-\left(\hat{p}-p\right)V+\left(\hat{\mu }-\mu \right)N,$$

or, as Hill wrote it (p. 24 in [20]),

(14) E=A+pV−μN,

with $A\equiv U-TS=\hat{\mu }N-\hat{p}V$. As the subsystem’s size approaches the macroscopic limit, the difference between $\left(\hat{p},\hat{\mu }\right)$ and $\left(p,\mu \right)$ vanishes, and, as a result U≈U≫E in every case.

### 3.2. Statistical Mechanics at Strong Coupling

Like in the previous section, we consider a small system with internal energy U, volume V and N particles. For simplicity, we constrain the system’s temperature to be *T*, while we allow the pressure and chemical potential to fluctuate as a result of interactions with the environment. The partition function $Z_{1}$ may be written using the Hamiltonian of mean force (7):

(15) $$Z_{1}=\sum_{i}e^{-H_{1}\left(i\right)/k_{B}T},$$

where, due to the nonvanishing interaction term in (7), $H_{1}\left(i\right)$ is temperature-dependent. In the following, and for notational simplicity, we drop the subindex 1 used to label the system in Equation (7). The partition function (15) may be used to find the internal energy (4) of the system, resulting in a expression of the form (9), with

(16) $$U\equiv \langle H(i)\rangle ,$$

and

(17) $$E\equiv -T\langle \frac{\partial H(i)}{\partial T}\rangle .$$

The quantity *U* is the reference energy for the bare system in the absence of coupling. The additional term E is an excess energy resulting from strong interactions with the environment, which cause the effective Hamiltonian to be temperature-dependent. When the interaction I is absent (isolated system) or negligible (large system), Equation (7) simplifies and the derivative in (17) becomes zero.

The system’s true pressure $\hat{p}$ is given by the usual

(18) $$\hat{p}=-\frac{\partial U}{\partial V},$$

or

(19) $$\hat{p}=p+\Delta p,$$

where p≡−∂U/∂V is the pressure in the absence of coupling, and

(20) $$\Delta p\equiv -\frac{\partial E}{\partial V}$$

is the additional pressure due to the interactions with the environment captured in E. Likewise, the chemical potential $\hat{\mu }$ is given by

(21) $$\hat{\mu }=\frac{\partial U}{\partial N},$$

or

(22) $$\hat{\mu }=\mu +\Delta \mu ,$$

where μ≡∂U/∂N is the chemical potential of the bare system, and

(23) $$\Delta \mu \equiv \frac{\partial E}{\partial N}$$

is the additional chemical potential resulting from interactions. Combining (20) and (23), we see that the additional pressure and chemical potential change the internal energy of the strongly coupled system by an amount

(24) E=−VΔp+NΔμ.

Expression (24) is equal to the subdivision potential (13) introduced by Hill for the canonical case using thermodynamic arguments. However, in contrast to Hill’s purely thermodynamic approach, the subdivision potential has now a thermostatistical interpretation in terms of the temperature-dependence of the effective Hamiltonian (17). The method of statistical mechanics at strong coupling has recently been used to analyze interfacial phenomena, easily producing laws shown to be valid at the nanoscale [3].

#### Generalization to Other Ensembles

While the strong coupling analysis above was done for a system subject to canonical constraints, a similar statistical mechanical analysis may be carried out for other environmental variables.

In the grand canonical ensemble, the system has a fixed chemical potential, and the energy adjustment due to strong coupling is simply given by the first term in the right hand side of (24), which is the same as expression (11) of Hill’s thermodynamic theory. Likewise, in the isothermal-isobaric ensemble, the pressure of the system is fixed, and the energy correction E due to strong coupling is given by the second term in the rhs of (24) alone, which is the same as expression (12) in Hill’s theory.

The strong coupling contribution in each of the three ensembles is different, making them nonequivalent. However, for large systems, the coupling term I in (7) becomes negligible with respect to the bare system’s Hamiltonian. As a result, the temperature dependence of the effective Hamiltonian vanishes, and, as expected, all ensemble descriptions become equivalent.

It should be noted that, in addition to the energy adjustment (17) needed to match the environment’s temperature *T*, in the grand-canonical ensemble, the open system must also exchange particles in order to match the surrounding chemical potential μ. Therefore, the Boltzmann factor in (15) must be corrected with $e^{\mu n(i)}$, where n(i) is the effective number of particles in the open system. A statistical mechanical analysis of this strongly coupled system results, not only in a modified energy U=U+E, but also a modified number of particles N consisting of the bare $\langle n(i)\rangle$, and an additional adjustment term $-T\langle \partial n(i)/\partial T\rangle$ resulting from the strong coupling.

Likewise, in the isothermal-isobaric ensemble, the small system must not only match the environmental temperature *T* by modifying its energy. It must also match the environmental pressure *p* by exchanging volume with its surroundings. Therefore, the Boltzmann factor in (15) must be augmented with $e^{-pV(i)}$, where V(i) are effective volume states. This results, not only in a modified energy U=U+E, but also a modified volume V consisting of two terms: the bare system’s volume $\langle V(i)\rangle$, and an additional term $-T\langle \partial V(i)/\partial T\rangle$ representing adjustments caused by strong coupling.

## 4. Concluding Remarks

**Figure 1:**
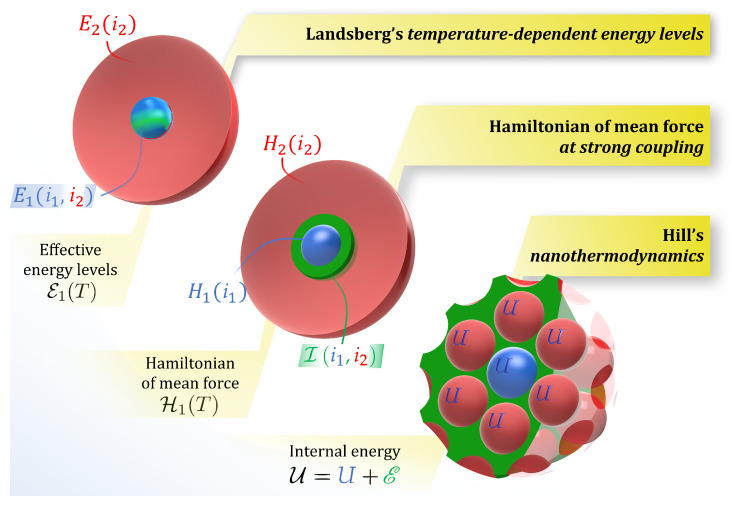
Illustration of the main features of the three small-system theories. In Landsberg’s theory, the energy levels $E_{1}$ of the system (blue sphere) are modified into $E_{1}$ due to its interaction with the (red) environment. Statistical mechanics at strong coupling starts with a total Hamiltonian consisting of the environment (red), the system (blue) and the interaction (green); averaging the system and the interaction over the environment results in a temperature-dependent Hamiltonian of mean force $H_{1}$. Hill’s nanothermodynamics considers the system of interest as if it were surrounded by a macroscopic set of interacting replicas; the interaction causes the effective energy to depart from *U* and become U.

The additivity of extensive quantities is an assumption upon which classical thermodynamics is based. However, this property does not apply to small systems [20] or, generally, to systems with sufficiently long-range interactions [1]. In this article, we have reviewed and shown the connection between three different theories proposed for the thermodynamic treatment of systems not satisfying the additivity ansatz. The differences and similarities between the three theories are illustrated in Figure 1.

Landsberg’s pioneering work consisted of assuming that when an otherwise isolated system comes into contact with a thermal bath, its energy levels are redistributed due to the interaction [6]. This results in an effective energy landscape that depends on the temperature of the bath, and it produces an extended thermostatistical framework that accounts for unknown interactions with the thermal environment (see Equations (4) and (5) above).

Years later, Hill proposed a thermodynamic framework for nonadditive systems by describing the mismatch between the internal energy of a small system when it is isolated vs. when it is embedded in a macroscopic ensemble of identical replicas. To account for this difference, he introduced the concept of subdivision potential, a new thermodynamic variable also known as *replica energy*. See [20,23] and Section 3.1 above.

More recently, a thermostatistical framework *at strong coupling* [30] has been formulated to account for the presence of the environment through a Hamiltonian of mean force (7). We show this modified Hamiltonian yields a temperature-dependent energy landscape as earlier suggested by Landsberg (see Equation (2)), and that it provides a thermostatistical foundation (17) for Hill’s subdivision potential E as it makes its appearance in the internal energy (9). Furthermore, we show that the thermostatistical treatment of the temperature-dependent Hamiltonian of mean force in the canonical ensemble results in (24), which is the subdivision potential (13) obtained by Hill. Hence, we assert that statistical mechanics at strong coupling is the bridge between Landsberg’s ’temperature-dependent energy levels’ and Hill’s nanothermodynamics. Indeed, and despite their independent inception and evolution, these three powerful theories describe similar phenomena, namely the nonadditivity caused by strong interactions.
